# Presynaptic GABA_B_ receptors inhibit vomeronasal nerve transmission to accessory olfactory bulb mitral cells

**DOI:** 10.3389/fncel.2023.1302955

**Published:** 2023-12-07

**Authors:** Jan Weiss, Frank Zufall

**Affiliations:** Center for Integrative Physiology and Molecular Medicine (CIPMM), Saarland University, Homburg, Germany

**Keywords:** GABA_B_ receptor, accessory olfactory bulb, olfactory plasticity, synaptic transmission, presynapse, pheromone

## Abstract

Vomeronasal sensory neurons (VSNs) recognize pheromonal and kairomonal semiochemicals in the lumen of the vomeronasal organ. VSNs send their axons along the vomeronasal nerve (VN) into multiple glomeruli of the accessory olfactory bulb (AOB) and form glutamatergic synapses with apical dendrites of mitral cells, the projection neurons of the AOB. Juxtaglomerular interneurons release the inhibitory neurotransmitter γ-aminobutyric acid (GABA). Besides ionotropic GABA receptors, the metabotropic GABA_B_ receptor has been shown to modulate synaptic transmission in the main olfactory system. Here we show that GABA_B_ receptors are expressed in the AOB and are primarily located at VN terminals. Electrical stimulation of the VN provokes calcium elevations in VSN nerve terminals, and activation of GABA_B_ receptors by the agonist baclofen abolishes calcium influx in AOB slice preparations. Patch clamp recordings reveal that synaptic transmission from the VN to mitral cells can be completely suppressed by activation of GABA_B_ receptors. A potent GABA_B_ receptor antagonist, CGP 52432, reversed the baclofen-induced effects. These results indicate that modulation of VSNs via activation of GABA_B_ receptors affects calcium influx and glutamate release at presynaptic terminals and likely balances synaptic transmission at the first synapse of the accessory olfactory system.

## Introduction

The accessory olfactory system is responsible for the detection and perception of conspecific and predator-derived chemical cues, including pheromones, and is known to initiate innate, stereotypical behaviors as well as experience-dependent social behaviors (Tirindelli et al., 2009; Chamero et al., 2012; Trouillet et al., 2021). The accessory olfactory bulb (AOB) receives sensory input from the vomeronasal organ, where vomeronasal sensory neurons (VSNs) recognize a variety of pheromones and kairomones (Leinders-Zufall et al., 2000; Papes et al., 2010; Isogai et al., 2011; Mohrhardt et al., 2018). Transmission of detected semiochemical signals along the vomeronasal nerve (VN) drives glutamate release onto postsynaptic projecting neurons, the mitral cells (Jia et al., 1999). VSN axons derived from VSNs expressing the same vomeronasal receptors terminate in numerous glomeruli, in contrast to the main olfactory bulb (MOB) (Belluscio et al., 1999; Rodriguez et al., 1999; Del Punta et al., 2002). Projections of apical and basal VSNs, expressing predominantly vomeronasal type 1 and type 2 receptors respectively, segregate into an anterior and a posterior domain of the AOB glomerular layer (GL) (Mohrhardt et al., 2018). Considerable differences exist in the structural composition of the AOB and MOB at the level of the external plexiform and the mitral/tufted cell layer (Salazar et al., 2006). AOB mitral cell bodies are dispersed and not organized in layers in the mitral cell layer (MCL). Their dendritic arbors target multiple glomeruli in the GL (Dulac and Wagner, 2006; Wagner et al., 2006). Mitral cell axons project to the medial amygdala, bed nucleus of the accessory olfactory tract, posterior medial cortical amygdala, and the bed nucleus of the stria terminalis and signals are further transmitted to hypothalamic nuclei (Brennan and Zufall, 2006). Processing and inhibition of mitral cell activity is provided by GABAergic juxtaglomerular cells and granule cells including reciprocal dendritic inhibition by ionotropic GABA receptors (Mohrhardt et al., 2018).

Presynaptic modulation of the first central synapse is widespread in sensory systems (Trussell, 2002; Chen and Regehr, 2003; Comitato and Bardoni, 2021). In the MOB, GABA_B_ and dopamine D2 receptors have been described to balance synaptic transmission at olfactory sensory neuron (OSN) terminals (McGann, 2013). Synaptic transmission at the first synapse of MOB and AOB sensory neurons relies on N-type calcium channels (Weiss et al., 2014), which are a prevalent target of presynaptic GABA_B_ receptors (Gassmann and Bettler, 2012; Delaney and Crane, 2016). GABA_B_ receptors consist of a ligand binding GABA_B1_ subunit and a G-protein activating GABA_B2_ subunit and are located at presynaptic axonal terminals and postsynaptic dendrites. Activation at presynaptic terminals leads to inhibition of voltage-gated calcium channels and hence calcium influx (Gassmann and Bettler, 2012). GABA_B_ receptor-mediated presynaptic inhibition is well established in axon terminals of OSNs in the main olfactory system (Aroniadou-Anderjaska et al., 2000; Wachowiak et al., 2005; Shao et al., 2009), but little is still known about the question whether similar mechanisms also operate in the accessory olfactory system. Whether modulation of VSN axon terminals is dependent on GABA_B_ receptor function has not been determined. Here, we set out to investigate this problem by using a combination of immunohistochemistry, confocal calcium imaging, and patch clamp recording in acute tissue slices from the mouse AOB. The results reveal that presynaptic inhibition of VSN nerve terminals through activation of GABA_B_ receptors provides a powerful mechanism for modulation of synaptic transmission at the first synapse of the accessory olfactory system.

## Materials and methods

### Mice

All procedures were approved by the Institutional Animal Care and Use Committee of the University of Saarland (UdS) School of Medicine and were in accordance with the laws for animal experiments issued by the German Government. Experiments were performed on 4–16-week-old mice of both sexes. Two different genotypes were used: (1) Wild-type mice (C57BL/6J, denoted as B6). (2) Mice expressing the calcium indicator GCamp3 in VSNs by crossing OMP-Cre mice (Li et al., 2004) (B6;129P2-Omp^TM4(cre)Mom^/MomJ) with the Ai38 floxed GCaMP3 reporter mouse line (Zariwala et al., 2012) (denoted as OMPcre:GCamp3). Mice were housed in microisolator cages on a 12:12 h light-dark cycle with food and water available *ad libitum*.

### Expression of GABA_B1_ receptor and vesicular glutamate transporter 2 (vGlut2)

Mice were deeply anesthetized with CO_2_, killed by decapitation, and OBs were rapidly dissected in ice-cold oxygenated (95% O_2_, 5% CO_2_) solution containing the following (in mM): 83 NaCl, 26.2 NaHCO_3_, 1 NaH_2_PO_4_, 2.5 KCl, 3.3 MgSO_4_, 0.5 CaCl_2_, 70 sucrose, pH 7.3 (osmolarity: 300 mOsm/l). The tissue was mounted on a vibratome (VT1000S; Leica Microsystems, Nussloch, Germany) and oblique-horizontal AOB slices (275 μm thick) were cut in the same solution. Slices were fixed in 4% (w/v) paraformaldehyde in PBS overnight. After washing free-floating slices were incubated in primary antibodies. Primary antibodies used were GABA_B1_ (1:500, mouse monoclonal, Cat. #ab55051, Abcam, Cambridge, UK) and vGlut2 (1:500, rabbit polyclonal, Cat. #135403, Synaptic Systems, Göttingen, Germany). Secondary antibodies were Alexa-Fluor 555 conjugated donkey-anti-mouse (1:1000), Alexa-Fluor 488 goat-anti-rabbit (1:1000, Invitrogen, Cat. # A-11034, Darmstadt, Germany). Free-floating slices were incubated in blocking solution (PBS pH 7.4, 4% normal horse serum, 0.5% Triton-X 100) for 1 h followed by primary antibody overnight at 4°C and secondary antibody for 2 h at room temperature. Fluorescence stacks were acquired on a Leica TCS SP5 II confocal laser scanning microscope (Leica Microsystems, Heidelberg, Germany), collapsed, and minimally adjusted in contrast and brightness using ImageJ (NIH).

### Calcium imaging

Oblique-horizontal AOB slices from OMPcre:GCamp3 mice were prepared for calcium imaging as well as electrophysiological patch clamp experiments. Slices were then placed in a Petri dish on an upright confocal laser scanning microscope (Leica TCS SP5 II, 20x water immersion objective HCX APO L20x/1.0w) equipped with Ar and He/Ne lasers. GCamp3 was excited at 488 nm and emission was measured between 490 and 560 nm. Images were acquired every 0.74 s (512 × 512). Baclofen, a GABA_B_ receptor agonist, and the potent GABA_B_ receptor antagonist CGP 52432 were applied to the AOB with a local perfusion system which produced a continuous solution stream along the AOB for 4–6 min, followed by a washout phase of 5–10 min. Interstimulus interval was 1–2 min. We stimulated the VN layer through a glass electrode (1–1.5 MΩ) filled with extracellular solution and connected to an Isolated Pulse Stimulator Model 2100 (A-M Systems Instruments, USA). We applied trains of electrical stimulations for 100 ms at a frequency of 100 Hz (each for 1 ms). All physiological measurements were carried out at room temperature.

Data analyses were performed using ImageJ (NIH) and Igor (Wavemetrics) software. Regions of interest (ROIs) were chosen in the glomerular layer by analysing the gray-scale images taken before and during activation. Any regions that exhibited signs of neuropil and had clear boundaries during activation were labeled as ROIs. Calcium responses of individual glomeruli were normalized to the resting fluorescence level of the glomerulus obtained before stimulation (ratio Fx/F0, Fx = actual fluorescence; F0 = mean fluorescence of the first 50 images of the experiment). Glomeruli were considered responsive if the deviation of fluorescence exceeded twice the SD of the mean of the baseline fluorescence noise and responded repeatedly to a stimulus. Amplitude peaks analyzed in all ROIs before baclofen/CGP 52432 treatment were averaged and normalized for each ROI. After baclofen/CGP 52432 application had reached a steady state effect, peaks were also determined, averaged, and normalized.

### Electrophysiology

Mice were deeply anesthetized with CO_2_, killed by decapitation, and OBs were rapidly dissected in ice-cold oxygenated (95% O_2_, 5% CO_2_) solution containing the following (in mM): 83 NaCl, 26.2 NaHCO_3_, 1 NaH_2_PO_4_, 2.5 KCl, 3.3 MgSO_4_, 0.5 CaCl_2_, 70 sucrose, pH 7.3 (osmolarity: 300 mOsm/l). Tissue was mounted on a vibratome (VT1000S; Leica Microsystems, Nussloch, Germany) and oblique-horizontal AOB slices (275 μm thick) were cut in the same solution. Slices were first stored in standard extracellular solution at 34–36°C for 30 min and then at room temperature until use. The extracellular solution contained the following (in mM): 125 NaCl, 25 NaHCO_3_, 2.5 KCl, 1.25 NaH_2_PO_4_, 1 MgCl_2_, 2 CaCl_2_ and 10 glucose (continuously bubbled with 95% O_2_, 5% CO_2_). Tissue slices were placed in the recording chamber and superfused at a rate of ∼2 ml/min (gravity flow) with sodium hydrogen carbonate-buffered extracellular solution bubbled with carbogen (95% O_2_, 5% CO_2_). Cells were visualized in intact tissue slices using a 40x water immersion objective lens (Olympus Optical) using infrared-optimized differential interference contrast optics and fluorescence illumination and a GFP filter set attached to the microscope to elucidate the morphology of Lucifer yellow-filled mitral cells (BX50WI, Olympus) (Weiss et al., 2014).

Slice patch clamp recordings were carried out at room temperature using an EPC-9 automated patch-clamp amplifier (HEKA Elektronik, Lambrecht, Germany) and Pulse 8.11 software as previously described (Weiss et al., 2014). Patch pipettes were pulled from borosilicate glass tubing (World Precision Instruments, Germany). Signals were filtered using an eight-pole Bessel filter built into the EPC-9 amplifier and digitized at a frequency ≥ filter cut-off frequency (VR-10B, Instrutech Corp.). The sampling rate for all recordings was 10 kHz. Recording pipettes had resistances of 3–6 MΩ. Positive pressure was applied when bringing the patch pipette toward the cell and negative pressure was applied for seal formation. Cells were voltage-clamped in the whole-cell patch-clamp mode. AOB mitral cells were located in the MCL of the AOB and were defined by their large (>10 μm) ellipsoidal- or triangular-shaped somata and their large glomerular dendrites targeting several glomeruli.

The intracellular solution contained (in mM): 140 CsCl, 1 EGTA, 10 HEPES, 2 ATP Na-salt, 1 GTP Mg-salt, 5 QX-314 (a lidocaine derivative), 0.1 Lucifer yellow; pH, 7.1; osmolarity, 290 mosm). Mitral cells were voltage clamped to −60 mV. Input resistances were approximately 600–1000 MΩ, series resistances were 7–20 MΩ and were left uncompensated in most recordings. The theoretical liquid junction potential between intracellular and extracellular compartments was calculated to be 4.1 mV and was not corrected.

After establishing a whole-cell recording, we waited at least 3 min before starting data acquisition to allow equilibration of the intracellular solution into the dendrites. The VN layer was stimulated through a glass electrode (1–1.5 MΩ) filled with extracellular solution and connected to an Isolated Pulse Stimulator Model 2100 (A-M Systems Instruments, USA). Stimulus intensity was typically 100 μA and the stimulus duration was 1 ms. Interstimulus interval was at least 40 s. In some experiments we applied a train of electrical stimulations for 100 ms at a frequency of 100 Hz (each for 1 ms). After two to three stimulations, the GABA_B_ receptor agonist baclofen (4 μM) was bath-applied for 3–4 min, followed by a washout period lasting at least 8 min. Bicuculline (5–10 μM) was used to block GABAergic inhibitory postsynaptic currents. Baclofen was prepared as concentrated aliquot and stored at 4°C. For each experiment, stock solutions were freshly diluted in extracellular solution. CGP 52432 was used at a concentration of 10 μM and was bath-applied for 6–7 min. All electrophysiological data were analyzed using Igor Pro software (WaveMetrics) and Excel (Microsoft). For pharmacological experiments, amplitudes of evoked excitatory postsynaptic currents (EPSCs) were assessed and each value was normalized to the value obtained by the second electrical stimulation.

Assumption of normality was tested before conducting the following statistical tests. Repeated measures ANOVA (rmANOVA) was used to measure the significance of differences between distributions, with the Bonferroni test as a *post hoc* comparison. If the dataset was not normally distributed, multiple groups were compared using Kruskal-Wallis one-way analysis of variance (ANOVA) with Mann-Whitney test as a *post hoc* comparison (*P < 0.05; **P < 0.01; ***P < 0.001). The probability of error level (alpha) was chosen to be 0.05. Unless otherwise stated, data are expressed as mean ± SEM. We used at least three different mice per experiment.

## Results

### GABA_B_ receptors are expressed in AOB glomeruli

**FIGURE 1:**
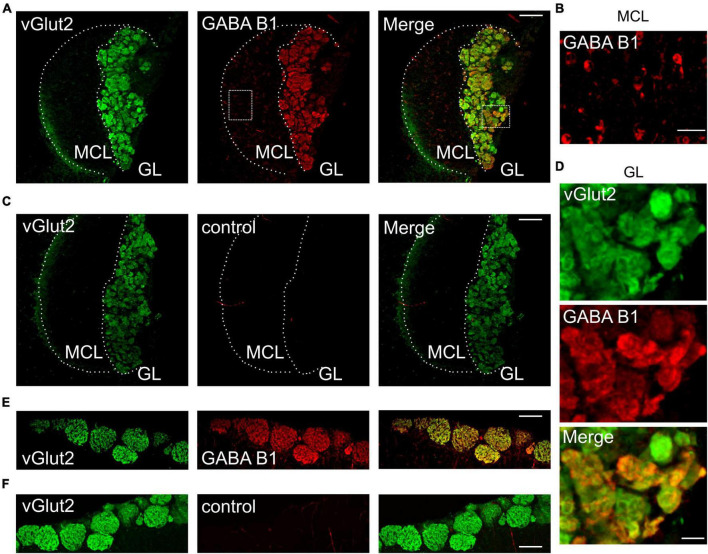
GABA_B1_ protein expression in the GL of the AOB colocalizes with vGlut2. **(A)** GABA_B1_ immunoreactivity (red) detected in the AOB (oblique-horizontal slice, 275 μm thick) of a B6 mouse is present in the GL and MCL. Immunoreactivity for the presynaptic marker vGlut2 (green) shows colocalization with GABA_B1_ in the GL, but not in the MCL. **(B)** GABA_B1_ immunoreactivity in the MCL at higher magnification showing numerous cell somata positive for GABA_B1_. **(C)** vGlut2 immunoreactivity and control experiment by omitting the primary GABA_B1_ antibody. GABA_B1_ immunoreactivity is absent (red), whereas vGlut2 (green) shows prominent staining in the GL. **(D)** Magnification of vGlut2 and GABA_B1_ immunoreactivity in the GL. Virtually all glomeruli are GABA_B1_ positive. **(E)** Control experiments showing GABA_B1_ immunoreactivity (red) in the GL of the MOB (horizontal slice, 275 μm) of a B6 mouse. Immunoreactivity for the presynaptic marker vGlut2 (green) shows colocalization with GABA_B1_ in the GL. **(F)** vGlut2 immunoreactivity and control experiment by omitting the primary GABA_B1_ antibody. MCL, mitral cell layer; GL, glomerular layer. Scale bars, 100 μm **(A,C,E,F)**; 25 μm **(B)**; 20 μm **(D)**.

GABA_B_ receptors play a major role in presynaptic modulation of OSNs in the main olfactory system (McGann, 2013). We hypothesized that GABA_B_ receptors may also be present at vomeronasal sensory nerve endings to modulate synaptic transfer at the first synapse of the accessory olfactory system. To assess GABA_B_ receptor expression in the AOB, we performed immunohistochemistry for the GABA_B1_ receptor subunit in oblique-horizontal AOB slices from adult male and female mice. We found strong GABA_B1_ immunoreactivity in virtually all AOB glomeruli (Figure 1A). We detected robust GABA_B1_ positive staining in the glomerular neuropil including VSN presynaptic boutons but not in the VN which consists of VSN axons (Figure 1A). GABA_B1_ expression was uniformly present in the anterior and posterior glomerular layers. Furthermore, somata of cells that presumably represent projecting neurons (mitral cells) in the MCL showed weak immunoreactivity and, therefore, could express GABA_B_ receptors (Figure 1B). No labeling was found in either the GL or the MCL after omission of the primary GABA_B_ antibody (Figure 1C) to control for non-specific antibody staining.

We performed double-labeling immunohistochemistry for GABA_B1_ and the vesicular glutamate transporter vGlut2 (Figure 1A), which is selectively expressed in presynaptic VSN axon terminals (Nakamura et al., 2005; Yokosuka, 2012). The results showed that GABA_B1_ and vGlut2 immunoreactivity colocalize in the GL of the AOB (Figure 1A), thus indicating the presence of GABA_B_ receptors at presynaptic axon terminals of VSNs. High magnification images of the glomerular layer demonstrated that all glomeruli express GABA_B1_, despite notable differences in fluorescence intensity (Figure 1D). Consistently, we also found characteristic protein expression of GABA_B1_ and vGlut2 in the glomerular neuropil of the MOB (Figures 1E, F).

Together, these results suggest that GABA_B1_ is likely to play an important role in modulating presynaptic transmitter release at the first synapse of the AOB.

### Imaging presynaptic calcium signals in AOB slices

**FIGURE 2:**
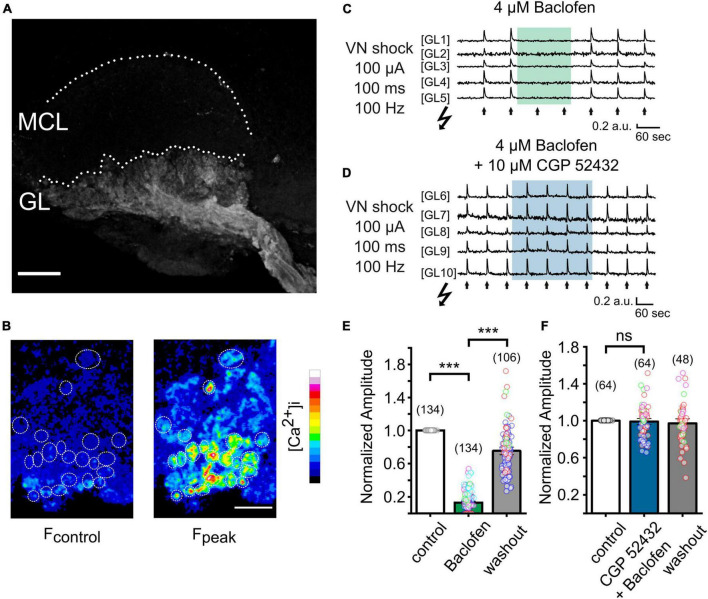
Imaging presynaptic calcium influx in VSN axon terminals *in vitro* in a OMPcre:GCamp3 mouse. **(A)** Collapsed stack of confocal images of an oblique-horizontal AOB slice prepared from a OMPcre:GCamp3 mouse. OMP-positive axons and axon terminals of VSNs are labeled by the calcium indicator GCamp3. GL, glomerular layer; MCL, mitral cell layer. **(B)** Images showing GCamp3 fluorescence at rest (F_control_) and at the peak of the response (F_peak_) to tetanic electrical stimulation (100 μA/100 ms/100 Hz) of the vomeronasal nerve (VN) layer in the GL. **(C)** Example traces obtained from single ROIs in the glomerular layer of the AOB (GL1 – GL5). VN shocks (arrows) repetitively induced GCamp3 calcium responses. Application of the GABA_B_ receptor agonist baclofen (4 μM) caused strong suppression of nerve-evoked calcium responses. **(D)** Example traces from single ROIs in the glomerular layer of the AOB (GL6 – GL10). When applied together with the GABA_B_ receptor antagonist CGP 52432 (10 μM), baclofen (4 μM) failed to suppress calcium responses in presynaptic terminals. **(E)** Group data showing normalized amplitudes to electrical stimulation of the VN before, during, and after treatment with baclofen (baclofen: 13 ± 1%, washout: 75 ± 3% of control; *n* = 5 mice; glomeruli of individual mice are color-coded). **(F)** Normalized amplitudes to electrical stimulation of the VN before, during, and after treatment with a combination of baclofen and CGP 52432 (CGP 52432 + baclofen: 99 ± 2%, washout: 97 ± 2% of control; *n* = 4 mice; glomeruli of individual mice are color-coded). Scale bars, 150 μm **(A)**; 50 μm **(B)**. The number of glomeruli is indicated in parentheses above each bar. ***P < 0.001.

**FIGURE 3:**
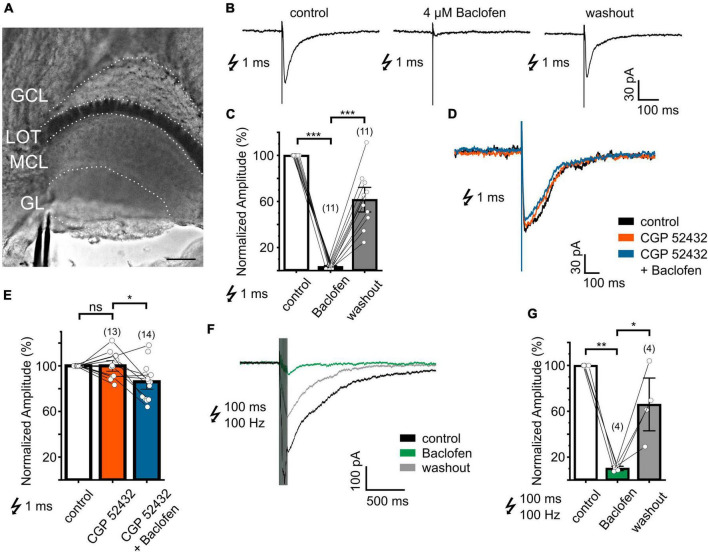
VN-evoked EPSCs recorded from AOB mitral cells are suppressed in a reversible manner by the GABA_B_ receptor agonist baclofen. **(A)** Micrograph of the AOB oblique-horizontal slice preparation (20x water immersion objective) showing the location of the stimulation pipette in the VN layer. GL, glomerular layer; MCL, mitral cell layer; LOT, lateral olfactory tract; GCL, granule cell layer. **(B)** Example of EPSCs of a mitral cell recorded before, during, and after treatment with baclofen (4 μM). **(C)** Group data showing the effect of baclofen (4 μM) on normalized EPSC peak amplitudes (baclofen: 3 ± 1%, washout: 62 ± 7% of control; *n* = 6 mice). **(D)** Superimposed EPSCs of a mitral cell recorded before and during treatment with CGP 52432 alone and in combination with baclofen. **(E)** Effect of CGP 52432 alone and CGP 52432 + baclofen on normalized EPSC peak amplitudes (CGP 52432: 100 ± 3%, CGP 52432 + baclofen: 84 ± 4% of control; *n* = 8 mice). **(F)** Time course of EPSCs of a mitral cell evoked by tetanic stimulation before, during, and after treatment with baclofen (4 μM). **(G)** Effect of baclofen (4 μM) on normalized EPSC peak amplitudes after tetanic stimulation (baclofen: 10 ± 1%, washout: 66 ± 13% of control; *n* = 3 mice). Scale bar, 150 μm **(A)**. The number of independent recordings is indicated in parentheses above each bar. *P < 0.05, **P < 0.01, and ***P < 0.001.

To examine the functional role of GABA_B_ receptors in the AOB, we next attempted to visualize the activation of VSN presynaptic terminals through spatiotemporal confocal calcium imaging. We generated OMPcre:GCamp3 mice in which GCamp3 is expressed in all OMP-positive sensory neurons, including VSNs. We first examined the expression pattern of GCaMP3 in VSNs. In Figure 2A, a collapsed confocal stack of a oblique-horizontal AOB slice shows exclusive GCamp3 expression in axons and synaptic terminals of VSNs. Using laser-scanning confocal microscopy, we imaged calcium signals in sensory neuron axon terminals in the GL of oblique-horizontal AOB slices. To investigate GABA_B_ receptor function underlying transmitter release from VN terminals, we performed time-lapse confocal imaging in response to tetanic electrical stimulation (10 pulses at 100 Hz, each pulse lasting 1 ms) of the VN layer (see Figure 3A). Electrical stimulation evoked a transient increase in GCamp3 fluorescence across the GL, indicative of calcium influx and transmitter release (Figures 2B, C). Glomerulus-like structures in the glomerular layer were identified as regions of interest (ROIs) and time-lapse calcium imaging data in these regions were analyzed. Fluorescence intensity considerably increased after electrical stimulation, as illustrated in Figure 2B.

### GABA_B_ receptor inhibition of presynaptic calcium influx

To determine the contribution of GABA_B_ receptors to presynaptic calcium signals at VSN axon terminals, we recorded fluorescence intensity to a saturating electrical stimulus (100 μA) before, during and after application of the potent and reversible GABA_B_ receptor agonist baclofen (4 μM) (Figure 2C). Baclofen dramatically reduced the peak amplitudes of calcium signals by 87 ± 1% (Figures 2C, E; 134 ROIs in 9 experiments; Kruskal–Wallis ANOVA, *P* < 0.001), an effect that was fully reversible. Calcium transients reappeared after several minutes of agonist washout (Figures 2C, E).

To confirm that the suppressing effect of baclofen on presynaptic calcium signals was mediated by GABA_B_ receptor activation, we next tested the ability of the selective GABA_B_ receptor antagonist CGP 52432 to reverse this effect. When baclofen was applied simultaneously with CGP 52432 (10 μM), the reduction of the calcium signal amplitude was nearly abolished (Figure 2D). Calcium transients remained stable during the application of the compounds and after washout (Figure 2F). After co-application of baclofen and CGP 52432, the normalized amplitude of fluorescence signals after tetanic stimulation was 99 ± 2% of control (Figure 2F, 64 ROIs in 4 experiments; rmANOVA: F (2, 94) = 0.43, *P* = 0.65).

Together, these results show that presynaptic calcium influx of VSN axon terminals can be strongly modulated by activation of GABA_B_ receptors, and this effect occurred throughout the AOB.

### Consequences for VN-evoked postsynaptic AOB mitral cell responses

AOB mitral cells are known to receive glutamatergic input from the VN (Jia et al., 1999), but unlike the MOB, AOB mitral cell dendrites target multiple glomeruli (Dulac and Wagner, 2006). Oblique-horizontal slicing of the AOB enabled us to perform patch clamp recordings from visually identified mitral cells located in the AOB MCL and to stimulate the VN layer with electrical shocks through an extracellularly placed glass electrode (Figure 3A).

To determine the impact of pharmacological manipulation of the GABA_B_ receptor on synaptic transmission, we recorded EPSCs from mitral cells and stimulated the VN with single brief (1 ms) or tetanic electrical shocks (10 pulses at 100 Hz, each pulse lasting 1 ms). Mitral cells were voltage-clamped at −60 mV and the intracellular solution contained the lidocaine derivative QX-314 to block voltage-gated sodium channels. The extracellular solution was supplemented with the GABA_A_ receptor antagonist bicuculline (5–10 μM) to prevent ionotropic GABAergic currents. Brief electrical stimulation evoked characteristic, transient inward currents with EPSC amplitudes ranging from −70 to −500 pA (Figure 3B, mean −247 ± 41 pA). We found that application of baclofen (4 μM) greatly diminished or even fully suppressed EPSC peak amplitudes to brief electrical stimulation (Figure 3B). In a total of 11 mitral cells, baclofen suppressed evoked amplitudes by 97 ± 1% (Figure 3C; rmANOVA: *F* (2, 20) = 143.6, *P* < 0.001). Washout of the GABA_B_ receptor agonist partially restored EPSCs (Figures 3B, C).

In the MOB, OSN presynaptic terminals are tonically inhibited by GABA_B_ receptors (Pírez and Wachowiak, 2008; Shao et al., 2009). When we applied the GABA_B_ receptor antagonist CGP 52432 to AOB slices, EPSC amplitudes of mitral cells did not differ significantly. Figure 3D depicts representative EPSC recordings before and during application of CGP 52432 (10 μM). In 13 mitral cells, EPSC amplitudes remained stable after CGP 52432 treatment (Figure 3E, 100 ± 3%; rmANOVA: *F* (2, 16) = 6.76, *P* < 0.001; Bonferroni *post-hoc* test: *P* = 0.99). These results suggest only a very minor contribution of tonic inhibition through GABA_B_ receptors at VSN axon terminals.

Similar to the calcium imaging experiments shown above, baclofen failed to efficiently suppress EPSC amplitudes when co-applied with CGP 52432 (Figure 3D). In 14 mitral cells, co-application resulted in amplitude reduction of 16 ± 4% (Figures 3D, E; Bonferroni *post hoc* test: *P* = 0.01), in stark contrast to the effect of baclofen alone (Figure 3D; 97% reduction). Thus, the GABA_B_ receptor agonist baclofen abolishes calcium influx at presynaptic VN terminals and prevents effective synaptic transmission to postsynaptic mitral cells, and this effect can be inhibited by CGP 52432.

Repetitive, prolonged stimulation of the VN can be considerably different from stimulation with brief pulses and presumably recruits additional calcium channels at the presynapse (Weiss et al., 2014). We performed tetanic stimulation (10 pulses at 100 Hz, each pulse lasting 1 ms) that evoked transient inward currents with amplitudes of −170 to −380 pA (mean: −283 ± 54 pA) in 4 mitral cells, comparable to EPSCs after brief stimulations. When we examined the effect of baclofen on mitral cell EPSCs evoked by tetanic stimulation, the amplitudes were still significantly reduced by 90 ± 1% after baclofen administration (Figures 3F, G; rmANOVA: *F* (2,6) = 24.7, *P* < 0.01; Bonferroni *post hoc* test: *P* < 0.01).

In summary, synaptic transmission from VN terminals to mitral cells can be effectively modulated by GABA_B_ receptor activation after single as well as tetanic stimulation, and this modulation seems to be mediated by presynaptic GABA_B_ receptors located at VSN axon terminals.

## Discussion

We investigated the role of GABA_B_ receptors in the AOB, the first relay station of information derived from the sensory epithelium of the VNO. We found that GABA_B1_ protein is abundantly expressed at VN terminals and is colocalized with the presynaptic vesicular glutamate transporter vGlut2. We also demonstrated that presynaptic calcium levels in VSN axon terminals increase after electrical stimulation of the VN, and that these calcium signals can be strongly suppressed by activation of GABA_B_ receptors. One major consequence of this presynaptic inhibition of calcium influx is a striking suppression of synaptic transmission from VSN terminals to postsynaptic mitral cells.

GABA_B_ receptors are heteromeric receptors composed of GABA_B1_ and GABA_B2_ subunits (Gassmann and Bettler, 2012) and suppress calcium influx at presynaptic terminals by inhibiting, for example, N-type calcium channels (Wachowiak et al., 2005). N-type calcium channels are the major contributors to presynaptic release in OSN and VSN terminals (Weiss et al., 2014). The expression of GABA_B1_ receptors in the MOB has been localized to the glomerular layer and to juxtaglomerular, mitral, and tufted cells (Bonino et al., 1999). A similar distribution has been described for the GABA_B2_ subunit (Panzanelli et al., 2004; Kratskin et al., 2006). We found strong immunoreactivity in the GL of the AOB that colocalized with an established marker of presynaptic terminals, vGlut2 (Nakamura et al., 2005; Figure 1). These results indicate abundant expression of GABA_B_ receptors at VSN terminals. Furthermore, cells in the MCL of the AOB (presumably mitral cells) were moderately stained by the GABA_B1_ antibody, consistent with previous results (Panzanelli et al., 2004) and reminiscent of the situation in the MOB (Bonino et al., 1999; Panzanelli et al., 2004), where GABA_B_ receptors are known to be involved in dendrodendritic inhibition (Isaacson and Vitten, 2003).

The vomeronasal sensory epithelium exhibits a dichotomous organization with apical G_αi2_+, V1r+ sensory cells projecting to the anterior AOB and basal G_αo_+, V2r+ sensory cells projecting to the posterior AOB (Mohrhardt et al., 2018). Both layers show considerable differences in ligand detection and behavioral functions (Chamero et al., 2011; Trouillet et al., 2019). Interestingly, expression of the GABA_B1_ subunit was uniformly distributed across the anterior and posterior AOB which indicates that GABA_B_ receptor function is likely to be crucial for processing of sensory information derived from both layers of the VNO.

In the MOB, optical imaging experiments measuring calcium signals or synaptic release in presynaptic terminals of OSNs revealed a decrease of ∼50% for calcium signals and almost 90% for transmitter release after baclofen application (McGann et al., 2005; Wachowiak et al., 2005; Vucinić et al., 2006; Pírez and Wachowiak, 2008). Baclofen reduced calcium transients in VSN terminals by 87% indicating a similar or even stronger effect on synaptic transmission at these synapses. With respect to electrophysiological postsynaptic responses, MOB synaptic transmission was also strongly inhibited by GABA_B_ receptor agonists, in the range of 50 to 80% (Nickell et al., 1994; Aroniadou-Anderjaska et al., 2000; Vaaga et al., 2017). Here, we found an even greater inhibition of synaptic transmission in the accessory olfactory system, with an almost complete loss of nerve-evoked EPSCs after activation of GABA_B_ receptors (Figure 3). Interestingly, GABA_B_ receptor-mediated presynaptic inhibition is more pronounced in pheromone-sensing OSNs compared to other olfactory channels in *Drosophila* (Root et al., 2008).

In previous experiments of the mammalian MOB, patch clamp recording and optical imaging revealed a considerable amount of tonic inhibition (Aroniadou-Anderjaska et al., 2000; Wachowiak et al., 2005; Pírez and Wachowiak, 2008; Shao et al., 2009), which has been proposed to depend on spontaneous bursting of external tufted cells (Shao et al., 2009). In our AOB experiments we found that presynaptic calcium signals as well as mitral cell EPSCs remained stable during treatment with GABA_B_ receptor antagonists. Tonic inhibition in the MOB relies heavily on bursting external tufted cells (Shao et al., 2009). Recently, a subpopulation of spontaneously and regularly bursting mitral cells has been described in the AOB (Vargas-Barroso et al., 2015; Gorin et al., 2016; Tsitoura et al., 2020), whose activity is highly dependent on extracellular calcium levels (Gorin et al., 2016). Our recording condition with 2 mM external calcium may have resulted in the inactivation of these oscillating AOB mitral cells, which may have shunted tonic inhibition. However, there are also important differences between the MOB and the AOB that influence the pattern of activity in the bulb. Whereas the MOB receives regular input during a sniff cycle, the VNO guides molecules into the vomeronasal lumen by active pumping (Levy et al., 2020), which could influence tonic inhibition. Further research, such as *in vivo* recordings, which uncovered the highest amount of tonic inhibition in the MOB (Pírez and Wachowiak, 2008), will be required to determine the contribution of tonic inhibition at VSN terminals.

GABA_B_ receptors have been implicated in aversive learning in the MOB (Okutani et al., 2003; Bhattarai et al., 2020). The vomeronasal system is known to initiate a variety of innate, pheromone-evoked behavioral responses (Mohrhardt et al., 2018) as well as specific forms of learned, experience-dependent behaviors (Brennan et al., 1990; Leinders-Zufall et al., 2004; Brennan and Zufall, 2006; Roberts et al., 2012; Hattori et al., 2017; Kaba et al., 2020; Trouillet et al., 2021). Modulation of synaptic transmission by GABA_B_ receptors at VSN terminals could potentially contribute to memory formation in the AOB. Furthermore, presynaptic inhibition is thought to extend the dynamic range of synaptic transmission (McGann, 2013). The non-uniform delivery of ligands into the vomeronasal lumen, which depends on the pumping process and temporal activation shifts between the anterior and posterior parts along the VNO, might require a high degree of dynamic range control in the accessory olfactory system.

In conclusion, we obtained new insights into the role of presynaptic GABA_B_ receptors in the mammalian accessory olfactory system. The evidence presented here suggests a central role of GABA_B_ receptors in presynaptic modulation of VSN terminals. Activation of these receptors abolished presynaptic calcium influx and synaptic transmission, thus revealing a powerful mechanism for the presynaptic control of information processing at the first synapse of the accessory olfactory system.

## Data availability statement

The raw data supporting the conclusions of this article will be made available by the authors, without undue reservation.

## Ethics statement

The animal study was approved by the Institutional Animal Care and Use Committee of the University of Saarland (UdS) School of Medicine. The study was conducted in accordance with the local legislation and institutional requirements.

## Author contributions

JW: Conceptualization, Investigation, Methodology, Visualization, Writing – original draft, Writing – review and editing. FZ: Funding acquisition, Supervision, Writing – review and editing.

## References

[B1] Aroniadou-Anderjaska V. Zhou F. M. Priest C. A. Ennis M. Shipley M. T. (2000). Tonic and synaptically evoked presynaptic inhibition of sensory input to the rat olfactory bulb via GABA(B) heteroreceptors. *J. Neurophysiol.* 84 1194–1203. 10.1152/jn.2000.84.3.1194 10979995

[B2] Belluscio L. Koentges G. Axel R. Dulac C. (1999). A map of pheromone receptor activation in the mammalian brain. *Cell* 97 209–220. 10219242 10.1016/s0092-8674(00)80731-x

[B3] Bhattarai J. P. Schreck M. Moberly A. H. Luo W. Ma M. (2020). Aversive learning increases release probability of olfactory sensory neurons. *Curr. Biol.* 30:e3. 10.1016/j.cub.2019.11.006 31839448 PMC6946845

[B4] Bonino M. Cantino D. Sassoè-Pognetto M. (1999). Cellular and subcellular localization of gamma-aminobutyric acid B receptors in the rat olfactory bulb. *Neurosci. Lett.* 274 195–198. 10.1016/s0304-3940(99)00697-7 10548423

[B5] Brennan P. Kaba H. Keverne E. B. (1990). Olfactory recognition: a simple memory system. *Science* 250 1223–1226. 2147078 10.1126/science.2147078

[B6] Brennan P. A. Zufall F. (2006). Pheromonal communication in vertebrates. *Nature* 444 308–315. 17108955 10.1038/nature05404

[B7] Chamero P. Katsoulidou V. Hendrix P. Bufe B. Roberts R. Matsunami H. (2011). G protein Gαo is essential for vomeronasal function and aggressive behavior in mice. *Proc. Natl. Acad. Sci. U.S.A.* 108 12898–12903. 10.1073/pnas.1107770108 21768373 PMC3150917

[B8] Chamero P. Leinders-Zufall T. Zufall F. (2012). From genes to social communication: molecular sensing by the vomeronasal organ. *Trends Neurosci.* 35 597–606. 22658923 10.1016/j.tins.2012.04.011

[B9] Chen C. Regehr W. G. (2003). Presynaptic modulation of the retinogeniculate synapse. *J. Neurosci.* 23 3130–3135. 12716920 10.1523/JNEUROSCI.23-08-03130.2003 PMC6742324

[B10] Comitato A. Bardoni R. (2021). Presynaptic inhibition of pain and touch in the spinal cord: from receptors to circuits. *Int. J. Mol. Sci.* 22:414. 10.3390/ijms22010414 33401784 PMC7795800

[B11] Del Punta K. Puche A. Adams N. C. Rodriguez I. Mombaerts P. (2002). A divergent pattern of sensory axonal projections is rendered convergent by second-order neurons in the accessory olfactory bulb. *Neuron* 35 1057–1066. 10.1016/s0896-6273(02)00904-2 12354396

[B12] Delaney A. J. Crane J. W. (2016). Presynaptic GABA B receptors reduce transmission at parabrachial synapses in the lateral central amygdala by inhibiting N-type calcium channels. *Sci. Rep.* 6:19255. 10.1038/srep19255 26755335 PMC4709695

[B13] Dulac C. Wagner S. (2006). Genetic analysis of brain circuits underlying pheromone signaling. *Annu. Rev. Genet.* 40 449–467. 16953793 10.1146/annurev.genet.39.073003.093937

[B14] Gassmann M. Bettler B. (2012). Regulation of neuronal GABA(B) receptor functions by subunit composition. *Nat. Rev. Neurosci.* 13 380–394. 22595784 10.1038/nrn3249

[B15] Gorin M. Tsitoura C. Kahan A. Watznauer K. Drose D. R. Arts M. (2016). Interdependent conductances drive infraslow intrinsic rhythmogenesis in a subset of accessory olfactory bulb projection neurons. *J. Neurosci.* 36 3127–3144. 10.1523/JNEUROSCI.2520-15.2016 26985025 PMC6705527

[B16] Hattori T. Osakada T. Masaoka T. Ooyama R. Horio N. Mogi K. (2017). Exocrine gland-secreting peptide 1 is a key chemosensory signal responsible for the bruce effect in mice. *Curr. Biol.* 27 3197–3201. 10.1016/j.cub.2017.09.013 29033330

[B17] Isaacson J. S. Vitten H. (2003). GABA(B) receptors inhibit dendrodendritic transmission in the rat olfactory bulb. *J. Neurosci.* 23 2032–2039. 12657661 10.1523/JNEUROSCI.23-06-02032.2003 PMC6742016

[B18] Isogai Y. Si S. Pont-Lezica L. Tan T. Kapoor V. Murthy V. N. (2011). Molecular organization of vomeronasal chemoreception. *Nature* 478 241–245. 21937988 10.1038/nature10437 PMC3192931

[B19] Jia C. Chen W. R. Shepherd G. M. (1999). Synaptic organization and neurotransmitters in the rat accessory olfactory bulb. *J. Neurophysiol.* 81 345–355. 9914294 10.1152/jn.1999.81.1.345

[B20] Kaba H. Fujita H. Agatsuma T. Matsunami H. (2020). Maternally inherited peptides as strain-specific chemosignals. *Proc. Natl. Acad. Sci. U. S. A.* 117 30738–30743. 10.1073/pnas.2014712117 33199615 PMC7720231

[B21] Kratskin I. Kenigfest N. Rio J. P. Djediat C. Reperant J. (2006). Immunocytochemical localization of the GABA B2 receptor subunit in the glomeruli of the mouse main olfactory bulb. *Neurosci. Lett.* 402 121–125. 10.1016/j.neulet.2006.03.077 16714082

[B22] Leinders-Zufall T. Brennan P. Widmayer P. Maul-Pavicic A. Jäger M. Li X. H. (2004). MHC class I peptides as chemosensory signals in the vomeronasal organ. *Science* 306 1033–1037. 10.1126/science.1102818 15528444

[B23] Leinders-Zufall T. Lane A. P. Puche A. C. Ma W. Novotny M. V. Shipley M. T. (2000). Ultrasensitive pheromone detection by mammalian vomeronasal neurons. *Nature* 405 792–796. 10.1038/35015572 10866200

[B24] Levy D. R. Sofer Y. Brumfeld V. Zilkha N. Kimchi T. (2020). the nasopalatine ducts are required for proper pheromone signaling in mice. *Front. Neurosci.* 14:585323. 10.3389/fnins.2020.585323 33328853 PMC7710809

[B25] Li J. Ishii T. Feinstein P. Mombaerts P. (2004). Odorant receptor gene choice is reset by nuclear transfer from mouse olfactory sensory neurons. *Nature* 428 393–399. 10.1038/nature02433 15042081

[B26] McGann J. P. (2013). Presynaptic inhibition of olfactory sensory neurons: new mechanisms and potential functions. *Chem. Senses* 38 459–474. 23761680 10.1093/chemse/bjt018 PMC3685425

[B27] McGann J. P. Pirez N. Gainey M. A. Muratore C. Elias A. S. Wachowiak M. (2005). Odorant representations are modulated by intra- but not interglomerular presynaptic inhibition of olfactory sensory neurons. *Neuron* 48 1039–1053. 10.1016/j.neuron.2005.10.031 16364906

[B28] Mohrhardt J. Nagel M. Fleck D. Ben-Shaul Y. Spehr M. (2018). Signal detection and coding in the accessory olfactory system. *Chem. Senses* 43 667–695. 30256909 10.1093/chemse/bjy061 PMC6211456

[B29] Nakamura K. Hioki H. Fujiyama F. Kaneko T. (2005). Postnatal changes of vesicular glutamate transporter (VGluT)1 and VGluT2 immunoreactivities and their colocalization in the mouse forebrain. *J. Comp. Neurol.* 492 263–288. 10.1002/cne.20705 16217795

[B30] Nickell W. T. Behbehani M. M. Shipley M. T. (1994). Evidence for GABA B-mediated inhibition of transmission from the olfactory nerve to mitral cells in the rat olfactory bulb. *Brain Res. Bull.* 35 119–123. 10.1016/0361-9230(94)90091-4 7953767

[B31] Okutani F. Zhang J. J. Otsuka T. Yagi F. Kaba H. (2003). Modulation of olfactory learning in young rats through intrabulbar GABA(B) receptors. *Eur. J. Neurosci.* 18 2031–2036. 10.1046/j.1460-9568.2003.02894.x 14622236

[B32] Panzanelli P. López-Bendito G. Luján R. Sassoé-Pognetto M. (2004). Localization and developmental expression of GABA(B) receptors in the rat olfactory bulb. *J. Neurocytol.* 33 87–99. 10.1023/B:NEUR.0000029650.28943.b2 15173634

[B33] Papes F. Logan D. W. Stowers L. (2010). The vomeronasal organ mediates interspecies defensive behaviors through detection of protein pheromone homologs. *Cell* 141 692–703. 20478258 10.1016/j.cell.2010.03.037 PMC2873972

[B34] Pírez N. Wachowiak M. (2008). In vivo modulation of sensory input to the olfactory bulb by tonic and activity-dependent presynaptic inhibition of receptor neurons. *J. Neurosci.* 28 6360–6371. 10.1523/JNEUROSCI.0793-08.2008 18562606 PMC2566846

[B35] Roberts S. A. Davidson A. J. Mclean L. Beynon R. J. Hurst J. L. (2012). Pheromonal induction of spatial learning in mice. *Science* 338 1462–1465. 10.1126/science.1225638 23239735

[B36] Rodriguez I. Feinstein P. Mombaerts P. (1999). Variable patterns of axonal projections of sensory neurons in the mouse vomeronasal system. *Cell* 97 199–208. 10.1016/s0092-8674(00)80730-8 10219241

[B37] Root C. M. Masuyama K. Green D. S. Enell L. E. Nässel D. R. Lee C. H. (2008). A presynaptic gain control mechanism fine-tunes olfactory behavior. *Neuron* 59 311–321. 10.1016/j.neuron.2008.07.003 18667158 PMC2539065

[B38] Salazar I. Sanchez-Quinteiro P. Cifuentes J. M. Fernandez De Troconiz P. (2006). General organization of the perinatal and adult accessory olfactory bulb in mice. *Anat. Record Part A Discov. Mol. Cell. Evol. Biol.* 288A 1009–1025. 10.1002/ar.a.20366 16892425

[B39] Shao Z. Puche A. C. Kiyokage E. Szabo G. Shipley M. T. (2009). Two GABAergic intraglomerular circuits differentially regulate tonic and phasic presynaptic inhibition of olfactory nerve terminals. *J. Neurophysiol.* 101 1988–2001. 10.1152/jn.91116.2008 19225171 PMC2695638

[B40] Tirindelli R. Dibattista M. Pifferi S. Menini A. (2009). From Pheromones to Behavior. *Physiol. Rev.* 89 921–956. 19584317 10.1152/physrev.00037.2008

[B41] Trouillet A. C. Keller M. Weiss J. Leinders-Zufall T. Birnbaumer L. Zufall F. (2019). Central role of G protein Gαi2 and Gαi2^+^ vomeronasal neurons in balancing territorial and infant-directed aggression of male mice. *Proc. Natl. Acad. Sci. U. S. A.* 116 5135–5143. 30804203 10.1073/pnas.1821492116 PMC6421405

[B42] Trouillet A.-C. Moussu C. Poissenot K. Keller M. Birnbaumer L. Leinders-Zufall T. (2021). Sensory detection by the vomeronasal organ modulates experience-dependent social behaviors in female mice. *Front. Cell. Neurosci.* 15:638800. 10.3389/fncel.2021.638800 33679330 PMC7925392

[B43] Trussell L. O. (2002). Modulation of transmitter release at giant synapses of the auditory system. *Curr. Opin. Neurobiol.* 12 400–404. 12139987 10.1016/s0959-4388(02)00335-5

[B44] Tsitoura C. Malinowski S. T. Mohrhardt J. Degen R. Dibenedictis B. T. Gao Y. (2020). Synchronous infra-slow oscillations organize ensembles of accessory olfactory bulb projection neurons into distinct microcircuits. *J. Neurosci.* 40 4203–4218. 10.1523/JNEUROSCI.2925-19.2020 32312886 PMC7244196

[B45] Vaaga C. E. Yorgason J. T. Williams J. T. Westbrook G. L. (2017). Presynaptic gain control by endogenous cotransmission of dopamine and GABA in the olfactory bulb. *J. Neurophysiol.* 117 1163–1170. 10.1152/jn.00694.2016 28031402 PMC5340883

[B46] Vargas-Barroso V. Ordaz-Sánchez B. Peña-Ortega F. Larriva-Sahd J. A. (2015). Electrophysiological evidence for a direct link between the main and accessory olfactory bulbs in the adult rat. *Front. Neurosci.* 9:518. 10.3389/fnins.2015.00518 26858596 PMC4726767

[B47] Vucinić D. Cohen L. B. Kosmidis E. K. (2006). Interglomerular center-surround inhibition shapes odorant-evoked input to the mouse olfactory bulb in vivo. *J. Neurophysiol.* 95 1881–1887. 10.1152/jn.00918.2005 16319205

[B48] Wachowiak M. Mcgann J. P. Heyward P. M. Shao Z. Puche A. C. Shipley M. T. (2005). Inhibition of olfactory receptor neuron input to olfactory bulb glomeruli mediated by suppression of presynaptic calcium influx. *J. Neurophysiol.* 94 2700–2712. 15917320 10.1152/jn.00286.2005 PMC1282456

[B49] Wagner S. Gresser A. L. Torello A. T. Dulac C. (2006). A multireceptor genetic approach uncovers an ordered integration of VNO sensory inputs in the accessory olfactory bulb. *Neuron* 50 697–709. 10.1016/j.neuron.2006.04.033 16731509

[B50] Weiss J. Pyrski M. Weissgerber P. Zufall F. (2014). Altered synaptic transmission at olfactory and vomeronasal nerve terminals in mice lacking N-type calcium channel Cav2.2. *Eur. J. Neurosci.* 40 3422–3435. 10.1111/ejn.12713 25195871

[B51] Yokosuka M. (2012). Histological properties of the glomerular layer in the mouse accessory olfactory bulb. *Exp. Anim.* 61 13–24. 22293668 10.1538/expanim.61.13

[B52] Zariwala H. A. Borghuis B. G. Hoogland T. M. Madisen L. Tian L. Zeng H. (2012). A Cre-dependent GCaMP3 reporter mouse for neuronal imaging in vivo. *J. Neurosci.* 32 3131–3141. 10.1523/JNEUROSCI.4469-11.2012 22378886 PMC3315707

